# Supplementary material for: Predicting visual function by interpreting a neuronal wiring diagram
Source: Nature. 2024 Oct 2;634(8032):113–23. doi: 10.1038/s41586-024-07953-5 (PMC11446822; doi:10.1038/s41586-024-07953-5)
Supplement: Supplementary file 6 — CRF and ERF predictions for individual TmY4 and TmY9 cells. Analogous to Supplementary Data 3, but for TmY target types. Shown are the top four monosynaptic pathways, the strongest pathway passing through each of the top ten intermediary types (ranking from Extended Data Fig. 7), and the trisynaptic pathway Tm1–TmY–Dm3–TmY (see the section entitled Prediction of spatial normalization). [file 41586_2024_7953_MOESM6_ESM.zip › DataS4/TmY9q/720575940645514263.pdf]

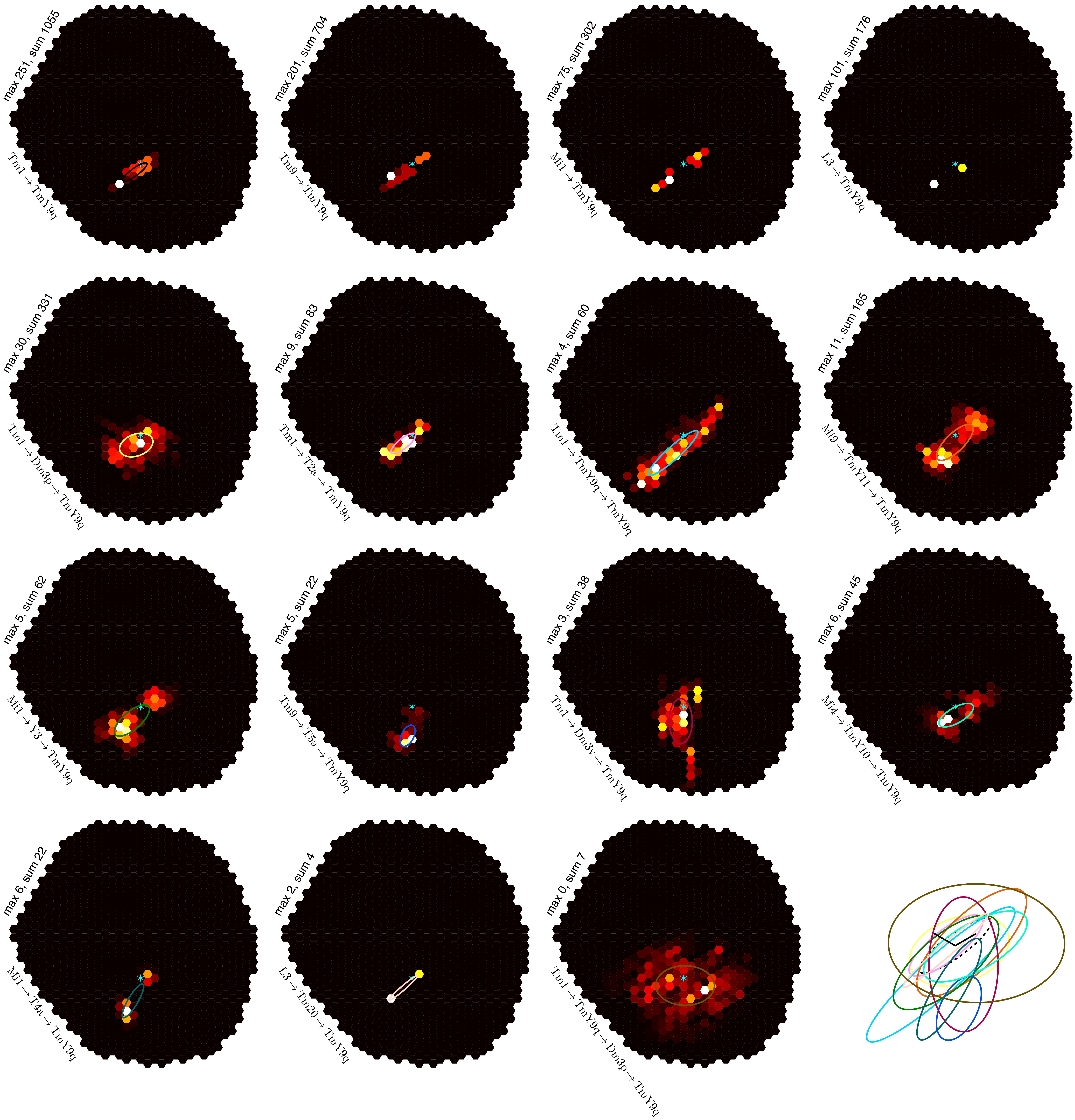

max 251, sum 1055
Tm1 → TmY9q
max 201, sum 704
Tm9 → TmY9q
max 75, sum 302
Mi1 → TmY9q
max 101, sum 176
L3 → TmY9q
max 30, sum 331
Tm1 → Dm3p → TmY9q
max 9, sum 83
Tm1 → T2a → TmY9q
max 4, sum 60
Tm1 → TmY9q → TmY9q
max 11, sum 165
Mi9 → TmY11 → TmY9q
max 5, sum 62
Mi1 → Y3 → TmY9q
max 5, sum 22
Tm9 → T5a → TmY9q
max 3, sum 38
Tm1 → Dm3v → TmY9q
max 6, sum 45
Mi4 → TmY10 → TmY9q
max 6, sum 22
Mi1 → T4a → TmY9q
max 2, sum 4
L3 → Tm20 → TmY9q
max 0, sum 7
Tm1 → TmY9q → Dm3p → TmY9q
